# Midterm outcomes of LAA occlusion with the AMPLATZER Cardiac Plug and AMPLATZER Amulet devices in a high-risk cohort

**DOI:** 10.1038/s41598-020-73381-w

**Published:** 2020-10-01

**Authors:** Tobias Tichelbäcker, L. Scherner, M. Puls, W. Schillinger, C. Adler, S. Baldus, C. Jacobshagen, M. Hünlich

**Affiliations:** 1 Department of Cardiology, Heart Center Cologne, Faculty of Medicine and University Hospital Cologne, University of Cologne, Cologne, Germany; 2 Heart Center Goettingen, University Medical Center Goettingen, Göttingen, Germany; 3 Vincentius-Diakonissen-Hospital Karlsruhe, Karlsruhe, Germany

**Keywords:** Cardiology, Interventional cardiology

## Abstract

LAA occlusion has become a favourable option in patients with atrial fibrillation not eligible for oral anticoagulation therapy. Proof of effectiveness of LAA closure devices in a midterm follow-up period. This retrospective single-center cohort study analysed outcome in patients treated with AMPLATZER Cardiac Plug or AMPLATZER Amulet device. A standardized follow-up by phone call focusing on data of death, stroke and bleeding events was performed. Routine antiplatelet strategy was DAPT for 3 months post procedural. 212 patients (mean age 77 ± 6 years) were included. Follow up was performed in 197 (93%) patients. Patients were at high risk for thromboembolic or bleeding events (prior stroke/TIA 29%; prior bleeding 54%. Overall, there was a mean follow-up period of 1244.2 days (± 756.7) and a total of 674 patient years. We observed 25 events later than day 8 post procedure. We were able to demonstrate a high effectiveness of the AMPLATZER Cardiac Plug/AMPLATZER Amulet devices regarding the prevention of stroke and bleedings in a high-risk real-world cohort during a midterm follow-up period. Overall, we observed remarkably lower rates of stroke and bleedings as predicted with CHA_2_DS_2_–VASc and HASBLED scores.

## Introduction

LAA occlusion with percutaneous catheter-delivered devices has become an alternative in the prevention of stroke in patients with atrial fibrillation and high bleeding risk (ESC Guidelines 2016: IIb/B recommendation)^1^.

Beyond the seminal RCTs of Apixaban^2^, Rivaroxaban^3^ and Dabigatran^4^, real world data of British patients showed a high proportion of discontinuation of DOACs, indicating the clinical need and relevance for alternative options in stroke prevention in many patients^5^.

Several devices of different manufacturers are available in the US and European market but only one device was tested in a randomized controlled trial (RCT) with a long-term follow-up period of 5 years and favourable results^6^.

Regarding the AMPLATZER Cardiac Plug (ACP)/AMPLATZER Amulet device (Abbott—Chicago, Illinois, US) there is only limited evidence outside of RCTs—a large multi-centre registry with an overall follow-up of 1349 patient years showed beneficial results regarding implantation and short term outcomes (mean average follow up = 13 months)^7^.

Validation of success of prophylactic interventions differ from therapeutical interventions especially in stroke prevention because no “instant” effect is expectable postprocedural. Due to the low annular risk of stroke even in patients with higher CHA_2_DS_2_–VASc Scores (e.g. CHA_2_DS_2_–VASc 4 = 8.5% annual stroke rate)^8^ and possible procedure related complications, success of stroke prevention with percutaneous devices has to be proven after a longer time period than 1 or 2 years (classic follow-up period of therapeutical interventions).

Aim of this study was to prove midterm outcomes of LAA occlusion with the ACP/AMPLATZER Amulet device and effectiveness regarding prevention of stroke and bleedings in a high-risk cohort.

## Methods

We conducted a retrospective single-centre cohort study. 212 patients were enrolled and a follow-up was performed in 197 patients. Follow-Up was done with standardized phone calls. The study was approved by the local ethics committee (Ethic committee Göttingen: 22/4/11) and was conducted according to ICH-GCP standards and the declaration of Helsinki—due to the retrospective character of the study, the need for informed consent was waived by our local ethic committee (Ethic committee Göttingen).

### Patients, procedure and post procedural phase

The presented cohort is a consecutive all-comers cohort. 212 patients were enrolled between 08/2009 and 04/2016.

All procedures were conducted by only two interventional cardiologists at a single high-volume centre in Germany. During the procedures all patients underwent analgosedation due to need of a transoesophageal echocardiography (TOE) probe for procedure imaging guiding. After implantation of the occluder patients were monitored at the normal ward. A transthoracic echocardiography was routinely performed 4–6 h following the procedure to exclude pericardial effusion on the ward. At the first postprocedural day correct position of the LAA occluder was verified with fluoroscopy.

Patients were provided with dual antiplatelet therapy (DAPT: ASS and clopidogrel) for 3 months, only in cases of very high bleeding risk the duration of DAPT was shortened to 1 month. After DAPT mono therapy with ASS or Clopidogrel was recommended by our institution.

### Follow-up

A standardized phone follow-up interview was performed with special attention to bleeding and stroke history. If patients reported events of bleedings or strokes, their general practitioner was contacted for detailed information (e.g. hospital reports). If only rudimental information could be provided (e.g. “stroke in May 2015”), event was “under” estimated (May 1st, 2015).

Definitions of endpoints and categorisation of e.g. bleedings were used like proposed by Tzikas et al. in the “Munich consensus paper”^9^.

### Data analysis

Data was analysed following a three-step procedure. First, patients were pooled according to their CHA_2_DS_2_–VASc and HASBLED score to ensure a minimum group size (see following section for details). Second, for each of these pooled groups, weighted averages of 1-year survival rates using data from Pisters et al. (2010) and Lip et al. (2010) were calculated to form a hypothetical population that matched the composed groups. Then, the 1-year survival rates of the hypothetical control group were expressed as monthly survival rates. The underlying assumption that survival rates did not differ over months and years is arguably conservative since the 1-year survival rate by Pisters et al. (2010) and Lip et al. (2010) is used for later years as well, thus the 1-year survival rates will most likely be too high for later years. In consequence, estimates should, if at all, be downwards biased. In the last step, the two survival curves of the patients enrolled in the study and the hypothetical population curve based on the 1-year survival rates by Pisters et al. (2010) and Lip et al. (2010) were drawn and p-values were calculated using the log-rank test.

## Results

Between 08/2009 and 04/2016, a total of 212 patients were treated with the ACP (n = 102) and the AMPLATZER Amulet device (n = 110). Only one patient died in the first seven days post procedure (not related to the procedure: cardiogenic shock and multi organ failure in a patient with dilative cardiac failure at intensive care unit) and we were able to perform a follow-up interview in 93% of the patients (n = 197/211) and had a total of 674 follow-up years (mean FU time of 3.3 years per patient).

### Baseline characteristics

Patients treated with the ACP and AMPLATZER Amulet device at our centre were at higher age (mean age 77 years ± 6) and at high risk for thromboembolic events [CHA_2_DS_2_–VASc > 2 in 95% of the patients (201/212)] and high risk for bleeding [HAS BLED ≥ 3 in 77% of the patients (163/212)].

All patients suffered from atrial fibrillation, mainly paroxysmal atrial fibrillation (47.2% (99/212). Renal failure (increase in creatinine level at baseline) was present in nearly a half of the patients [57.5% (122/212)], other frequent comorbidities were arterial hypertension [89.6% (190/212)], diabetes [23.1% (49/212)] and coronary artery disease [59.4% (126/212)].

Nearly a third of the population had a prior stroke or TIA [28.8% (61/212)] (Table 1).

**Table 1:** Baseline characteristics.

Age (years)	77 ± 6
Body Mass Index	27 ± 5
Permanent AF, n (%)	81 (38.2)
Persistent AF, n (%)	31 (14.6)
Paroxysmal AF, n (%)	100 (47.2)
Art. hypertension	190 (89.6)
Diabetes, n (%)	49 (23.1)
Coronary artery disease, n (%)	126 (59.4)
Prior myocardial infarction, n (%)	52 (24.5)
Prior PCI, n (%)	70 (33)
Prior CABG, n (%)	33 (15.5)
Prior stroke/TIA, n (%)	61 (28.8)
Renal failure, n (%)	122 (57.5)

### Indications for LAA occlusion

Main indications for percutaneous LAA occlusion in our cohort were previous major bleeding (58%), inability to take anticoagulation (22%) and increased risk for bleeding (22%). Regarding previous major bleeding, gastrointestinal and intracranial bleeding could be identified as the main causes. An increased risk for bleeding was estimated in patients with recurrent falls with head trauma and significant musculoskeletal injury and in patients with a need of additional dual antiplatelet therapy in case of coronary artery disease and stenting.

### Successful implantation

According to the “Munich consensus document”^9^, successful implantation was subdivided into device success, technical success and procedural success.

We were able to reach a device success (device deployed and implanted in correct position) in all of our patients. Technical success was achieved in 99% due to one device embolization, whereas a procedural success was obtained in 94% of our patients. Main reasons for failure of a procedural success were minor access complications (e.g. drop in Hb values without need for transfusion or haematoma at access site) and harmless pericardial effusions.

### Stroke prevention

We observed a total of 11 strokes, three TIA episodes and one thromboembolic event (Thrombus in left atrium, no real “embolic” event). Within 674 patient years we observed 15 events resulting in a risk rate of 2.2 thromboembolic events per year. The calculated risk rate by CHA_2_DS_2_–VASc Scores was 5.9, so we were able to state a risk reduction of TE events by LAA occlusion of 61%.

**Figure 1:**
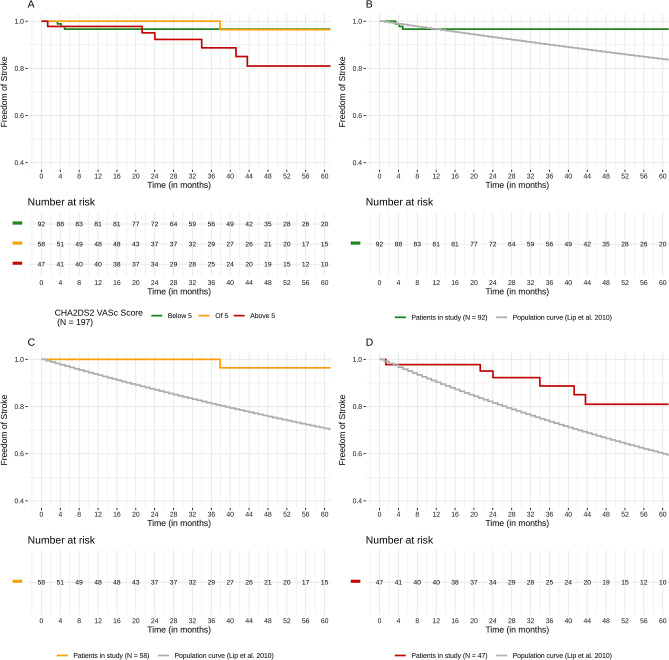
Kaplan–Meier survival estimates for stroke, TIA and thromboembolic events. Upper left: Kaplan–Meier curves of stroke events between the three risk groups (CHA_2_DS_2_–VASc of < 5, of and > 5. Upper right: Kaplan–Meier curves of observed stroke events in the CHA_2_DS_2_–VASc of < 5 and predicted with CHA_2_DS_2_–VASc (matched groups). Lower left: Kaplan–Meier curves of observed stroke events in the CHA_2_DS_2_–VASc of 5 and predicted with CHA_2_DS_2_–VASc (matched groups). Lower right: Kaplan–Meier curves of observed stroke events in the CHA_2_DS_2_–VASc of > 5 and predicted with CHA_2_DS_2_–VASc (matched groups).

We assigned all patients to their expected stroke risk calculated by CHA_2_DS_2_–VASc Scores and build three risk groups: CHA_2_DS_2_–VASc < 5 (n = 92), of 5 (n = 58) and above 5 (n = 47). In a second step we compared real-world data with a designed Kaplan–Meier curve according to the risk groups and consecutive stroke risk (Fig. 1). In all of the three risk groups we observed a significant lower stroke rate than estimated with the CHA_2_DS_2_-VASc Score (CHA_2_DS_2_–VASc below 5 p = 0.035; CHA_2_DS_2_–VASc of 5 p = 0.00084; CHA_2_DS_2_–VASc above 5 p = 0.017).

### Bleedings

**Figure 2:**
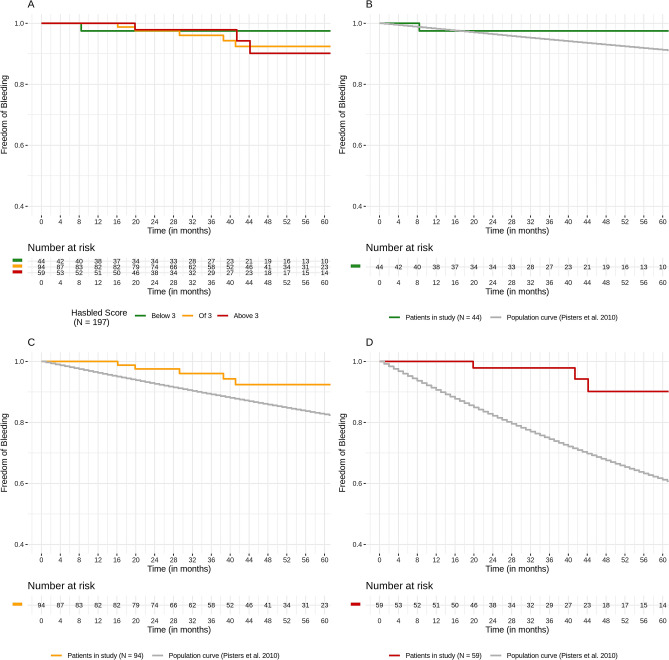
Kaplan–Meier survival estimates for bleedings. Upper left: Kaplan–Meier curves of bleeding events between the three risk groups (HAS-BLED score of < 3, 3 and > 3). Upper right: Kaplan–Meier curves of observed bleeding events in the HAS-BLED of < 3 and predicted with HAS-BLED score (matched groups). Lower left: Kaplan–Meier curves of observed bleeding events in the HAS-BLED of 3 and predicted with HAS-BLED score (matched groups). Lower right: Kaplan–Meier curves of observed bleeding events in the HAS-BLED of > 3 and predicted with HAS-BLED score (matched groups).

9 patients experienced a bleeding event after implantation of the LAA occlusion device. Analog to the stroke events we assigned each patient to their bleeding risk calculated by the HAS-BLED score. We build three groups with a HAS-BLED of below 3 (n = 44), of 3 (n = 94) and above 3 (n = 59) and compared their actual bleeding events with the calculated risk rates (Fig. 2). In the groups with HAS-BLED of 3 or above, significant lower bleeding rates as expected could be observed. Only in the group of HAS-BLED score below 3, just a trend of lower bleeding rates was noticed.

None of these bleeding events occurred in the first 3 months post implantation while dual antiplatelet therapy. One fatal (intracranial) bleeding was observed when a patient was incidentally set on anticoagulation. Of the 8 bleeding events, one more bleeding event was of intracranial origin, six bleedings had a gastrointestinal origin.

### Comparison of ACP and AMPLATZER AMULET devices

Two devices were used in the study. The first 102 patients were provided with the ACP device, the following 110 patients were treated with the AMPLATZER AMULET device. Stroke events in the ACP and AMPLATZER AMULET device differ between both groups with a trend towards the ACP group without reaching statistical significance (8/102 vs 3/110, p = 0.1) whereas the few TIA events similar occurred in the both groups (1/102 vs 2/110). A single thromboembolic event (thrombus formation in left atrium) was seen in the ACP group.

## Discussion

The presented retrospective real-world cohort of patients is, to our knowledge, one of the largest single-centre cohorts of patients treated with the ACP and AMPLATZER Amulet LAA occluder devices. Regarding the follow-up period this cohort has the first mid term results of the ACP and AMPLATZER Amulet devices with a mean FU of 3.3 years and a total of 674 follow-up years.

The presented results are comparable with the two multicentre registries of ACP and AMPLATZER Amulet devices: Tzikas et al.^7^ analysed a cohort of 1001 patients with 1349 patient years (mean FU 1.3 years) and reported a risk reduction of 59%. Berti et al.^10^ showed a risk reduction of 66% in 542 patients with a mean FU of 1.7 years. The most recent publication of a trial with only AMPLATZER Amulet devices implanted indicated a risk reduction of 60% in 950 patients with a total of 879 patient years (mean FU 0.9 years).

Patients treated in this study were at high risk for stroke and bleeding events due to a mean CHA_2_DS_2_–VASc of 5 (indicating an annular stroke risk of 6.7%) and mean HAS-BLED score of 3.

A significant stroke reduction rate was found and with 61% it is comparable to reduction rates of other published ACP/AMPLATZER Amulet cohorts^7,10,11^—this work firstly showed consistent results at midterm follow-up and at all risk groups.

All of our patients would not have been suitable for oral anticoagulation neither with warfarin nor with a DOAC due to their high bleeding risk. Bleeding complications under DAPT in the first 3 months followed by ASS or Clopidogrel monotherapy occurred in a remarkably low number of patients.

There were no bleedings under 3 months of DAPT therapy following the LAA-Occluder intervention supporting the safety of this concept in a high-risk cohort. One fatal bleeding occurred when a patient was incidentally provided with anticoagulation therapy in another hospital indicating the high risk for bleeding and continued oral anticoagulation in the present cohort.

The study was not powered and conducted to compare the ACP device with the AMPLATZER Amulet device, however a trend towards higher rates of strokes in the ACP group was observed. The only thromboembolic event with proof of a thrombus formation in the left atrium was proved in the ACP group. These findings may be caused by the improvements of the AMPLATZER Amulet in comparison to the ACP device (e.g. inverted attached screw on the proximal disc)^12^.

Trials for evaluation of interventions for stroke prophylaxis have to be conducted as midterm analysis (at least longer than 2 years) in order to obtain accurate results: In contrast to oral anticoagulation there are periprocedural risks that may favour medical treatment in comparative trials with a short follow-up. Furthermore, there is a difference to other percutaneous devices such as TAVI or MitraClip because no instant effect is measurable. The effect of this intervention is the absence of adverse events over time and therefor it can only be evaluated correctly with mid- to long-term follow-ups.

### Study limitations

The presented data contains single-centre non-randomized observations of patients treated with the ACP and AMPLATZER Amulet devices and results have to be interpreted with caution due to known limitations of single-centre studies. The findings are exploratory as the study is not powered to assess endpoints unequivocally. Furthermore, no clinical or echocardiographic follow-up was performed, nevertheless the authors are convinced that the endpoints of stroke and bleedings can be reliably reported by patients.

## Conclusion

The present study contains midterm data of LAA occlusion with ACP and AMPLATZER Amulet devices in a high-risk real-world cohort. We observed significantly lower stroke and bleeding rates than calculated with CHA_2_DS_2_–VASc Score and HAS-BLED score.

## Abbreviations

ACP: Amplatzer Cardiac Plug
CABG: Coronary artery bypass graft
DAPT: Dual antiplatelet therapy
DOAC: Direct oral anticoagulation
ESC: European Society of Cardiology
FU: Follow-up
LAA: Left atrial appendage
PCI: Percutaneous coronary intervention
TE: Thromboembolic
TIA: Transient ischemic attack
TOE: Transoesophageal echocardiography
